# Identification of Extracellular Actin As a Ligand for Triggering Receptor Expressed on Myeloid Cells-1 Signaling

**DOI:** 10.3389/fimmu.2017.00917

**Published:** 2017-08-07

**Authors:** Lei Fu, Li Han, Caiyun Xie, Wenke Li, Lan Lin, Shan Pan, You Zhou, Zhi Li, Meilin Jin, Anding Zhang

**Affiliations:** ^1^State Key Laboratory of Agricultural Microbiology, College of Veterinary Medicine, Huazhong Agricultural University, Wuhan, China; ^2^The Cooperative Innovation Center for Sustainable Pig Production, International Joint Research Center for Animal Disease Control, Wuhan, China; ^3^Key Laboratory of Development of Veterinary Diagnostic Products, Ministry of Agriculture, Wuhan, China

**Keywords:** sepsis, triggering receptor expressed on myeloid cells-1, actin, ligands, interaction, signaling

## Abstract

Triggering receptor expressed on myeloid cells-1 (TREM-1) is a potent amplifier of pro-inflammatory innate immune reactions, and it is an essential mediator of death in sepsis. However, the ligand for TREM-1 has not been fully identified. Previous research identified a natural ligand of TREM-1 distributed on platelets that contributed to the development of sepsis. However, the exact signal for TREM-1 recognition remains to be identified. Here, we identified actin as a TREM-1-interacting protein on platelets and found that recombinant actin could interact with recombinant TREM-1 extracellular domain directly. Furthermore, actin co-localized with TREM-1 on the surface of activated mouse macrophage RAW264.7 cells interacting with platelets. In addition, recombinant actin could enhance the inflammatory response of macrophages from *wt* mice but not from *trem1^−/−^* mice, and the enhancement could be inhibited by LP17 (a TREM-1 inhibitor) in a dose-dependent manner. Importantly, extracellular actin showed co-localization with TREM-1 in lung tissue sections from septic mice, which suggested that TREM-1 recognized actin during activation in sepsis. Therefore, the present study identified actin as a new ligand for TREM-1 signaling, and it also provided a link between both essential regulators of death in sepsis.

## Introduction

Sepsis is a progressive systemic inflammatory response syndrome associated with multiorgan dysfunction caused by overwhelming infection^1^. Despite decades of research and advances in clinical management, morbidity and mortality from sepsis remain substantial and have shown only modest improvements (1), and it is still the leading cause of death in critical care units (2). Because sepsis is viewed as an excessive host response inducing a complex network of molecular cascades leading to tissue damage, organ failure, and death (3), the activators of the innate immune system are recognized as possible treatment targets during sepsis. Damage-associated molecular patterns (DAMPs) are a kind of activator and contribute to the essential development of sepsis. Among these DAMPs, extracellular actin is associated with various severe inflammation (4), but it remains to be elucidated how extracellular actin activates the inflammatory signaling.

Triggering receptor expressed on myeloid cells-1 (TREM-1) belongs to the Ig superfamily and is an activating receptor that is expressed on myeloid cells (5). It can be induced at high levels on neutrophils and monocytes and further amplifies Toll-like receptor-initiated responses against microbial challenges, potentiating the secretion of pro-inflammatory cytokines with the help of the DAP12 adaptor protein in response to bacterial and fungal infections (6–8). Due to the key role of TREM-1 in amplifying the inflammatory response, TREM-1 was identified as an essential regulator of innate immunity in sepsis syndrome (9, 10), and it was confirmed to be an attractive target for the treatment of sepsis (6, 11–14). The natural ligand for TREM-1 is present on platelets (15), which is indispensable for regulating inflammatory processes such as sepsis (16). However, the ligand for TREM-1 on platelets remained to be characterized, although HMGB1 and PGLYRP1 were identified as ligands of the pathways (17, 18).

To this end, we have identified actin as a TREM-1-interacting protein on platelets, and the interaction is direct. Furthermore, actin co-localized with TREM-1 on the surface of the activated macrophages interacting with platelets, and it also enhanced the inflammatory response through TREM-1 signaling. Importantly, extracellular actin could be recognized by TREM-1 during sepsis. Therefore, extracellular actin is a new ligand for TREM-1 during sepsis, and the present study provides a link between both essential regulators of death in sepsis.

## Materials and Methods

### Ethics

The study was performed in strict accordance with the Guide for the Care and Use of Laboratory Animals Monitoring Committee of Hubei Province, China, and the protocol was approved by the Committee on the Ethics of Animal Experiments at the College of Veterinary Medicine, Huazhong Agricultural University (permit number: HZAUMO-2015-018). All efforts were made to minimize the suffering of the animals used in the study.

### Purification of Platelets

Platelets were isolated as described by Haselmayer et al. (15). Citrated mouse blood was centrifuged at 180 *g* for 15 min at room temperature to obtain platelet-rich plasma, which was further layered on 34% (wt/vol) BSA and centrifuged at 550 *g* for 10 min. Purified platelets were collected from the interphase and washed three times with HEPES buffer (5 mM HEPES, 137 mM NaCl, 2.7 mM, KCl, 11.9 mM NaHCO3, 1 mM MgCl, 0.1% BSA, 1% glucose, and 5 mM EGTA). If the platelets were used to active TREM-1 signaling, they would be fixed with 2% (wt/vol) paraformaldehyde (Sigma-Aldrich); some platelets were lysed with RIPA buffer [50 mM Tris-HCl (pH 8.0), 150 mM NaCl, 1%NP-40, 1 mM PMSF, and 1 mM EDTA] for extraction of total proteins.

### Preparation of Recombinant Extracellular Domain of Mouse TREM-1 (rTREM-1) and Recombinant Mouse β-Actin (rACTIN)

The rTREM-1 was prepared according to a previously published procedure (19) and then labeled with Cy5.5-NHS-Ester (Lumiprobe) for FACs analysis.

The coding sequence of mouse β-actin was cloned into pET28a and transformed into *Escherichia coli* (BL21) for expression. The rACTIN, which was fused with a 6His tag, was purified with Ni-nitrilotriacetic acid (NTA) agarose (Qiagen). Before rACTIN was used as a ligand of TREM-1 signaling, the protein was repeatedly treated with Triton X-114 to remove endotoxin (<0.001 EU/μg of protein).

### Flow Cytometry Analysis

C57BL/6 mice were purchased from the Laboratory Animal Center of Hubei Province (permit number: 42000600007246). Five 6-week-old female C57BL/6 mice were intravenously injected with LPS (5 mg/kg) via tail vein, and another five mice were sham treated with PBS as a control. Citrated blood was collected at 8 h post-injection and then treated with RBC Lysis Buffer (BioLegend). Prior to staining for flow cytometry analysis, the Fc receptors were blocked with rat anti-mouse CD16/32 (BioLegend). The cells were then stained with allophycocyanin-conjugated anti-mouse F4/80 (BioLegend), Percp/cy5.5-conjugated anti-mouse Ly-6G (BioLegend), and phycoerythrin (PE)-conjugated rat IgG2A anti-mouse TREM-1 (R&D systems) or PE-conjugated Rat IgG2a, κIsotype Ctrl Antibody (BioLegend) to analyze which cells expressed surface TREM-1.

In addition, the blood cells were also stained with Cy5.5-NHS-Ester labeled rTREM-1, PE/Cy7 conjugated anti-mouse CD41 (BioLegend) and PE-conjugated anti-mouse ly-6G (BioLegend) to analyze which cells expressed ligands of TREM-1. Subsequently, the cells were analyzed with the BD FACSVerse^TM^ Flow Cytometer and Flowjo 7.6.1 software.

### Identification and Characterization of TREM-1-Interacting Proteins

The rTREM-1 and the extracted total platelet proteins were incubated for 2 h at 4°C and then loaded onto NTA agarose for 1 h at 4°C. After washing with PBS five times, the proteins were eluted with 0.5 ml PBS buffer containing 250 mM imidazole and then further loaded onto ANTI-FLAG M2-Agarose Affinity Gel (Sigma) and incubated for 2 h at 4°C. After washing five times, the purified proteins were eluted with 100 µl of 0.1 M glycine. The purified proteins were further analyzed by SDS-PAGE, and the suspected rTREM-1-interacting proteins were excised from the gel slice and subjected to analysis by LC-MS/MS according to the procedure described previously (20).

To confirm the identification of TREM-1-interacting proteins, the purified proteins were further subjected to immunoblot analysis with rabbit anti-beta actin polyclonal antibody (Proteintech) and peroxidase-labeled goat anti-rabbit IgG (H + L) (KPL) or with mouse TREM-1 antibody antigen affinity-purified polyclonal goat IgG and peroxidase-labeled rabbit anti-goat IgG (H + L) (KPL), followed by development with SuperSignal^®^ West Femto Trial Kit (Thermo Scientific). Images were obtained on the MF Chem BIS Bio-Imaging System (DNR). All experiments were done in triplicate. To further confirm whether the protein interaction was direct, the purified rTREM-1 (fusion with 3FLAG and 6His Tag) and rACTIN (fusion with 6His Tag) were incubated for 1 h at 4°C and then loaded onto ANTI-FLAG M2-Agarose affinity Gel (Sigma) and incubated for 2 h at 4°C. After washing five times, the purified proteins were eluted with 100 µl of 0.1 M glycine. The purified proteins were then subjected for immunoblotting with rabbit anti-beta actin polyclonal antibody (Proteintech) and peroxidase-labeled goat anti-rabbit IgG (H + L) (KPL) or with mouse TREM-1 antibody antigen affinity-purified polyclonal goat IgG and peroxidase-labeled rabbit anti-goat IgG (H + L) (KPL).

### Analysis of the Co-Localization of TREM-1 and Actin by Laser Scanning Confocal Microscopy

Mouse macrophage RAW264.7 cells were treated with LPS (100 ng/ml) for 5 h and then incubated with platelets (platelets:RAW264.7 = 30:1) and/or rACTIN (1 µg/ml) for additional 3 h. RAW264.7 cells were mock treated with PBS as a control. All cells were washed with PBS for five times and then blocked with donkey sera (Proteintech). Subsequently, these cells were fixed with 2% (wt/vol) paraformaldehyde (Sigma-Aldrich) and then incubated with mouse TREM-1 antibody antigen affinity-purified polyclonal goat IgG (R&D) and rabbit anti-beta actin polyclonal antibody (Proteintech), followed by fluorescein (FITC)-conjugated affinipure donkey anti-rabbit IgG (H + L) (Proteintech) and CY3-conjugated affinipure donkey anti-goat IgG (H + L) (Proteintech). Cell nuclei were stained with Hoechest 33258 (Beyotime). After washing five times, all slides were mounted with 50% glycerol and covered with glass cover slips, followed by analysis with a LSM880 with Airyscan laser scanning confocal microscope (ZEISS) and ZEN 2.3 LITE software (ZEISS). 405, 488, and 561 nm wavelengths were used because the Excitation/Emission wavelengths of Hoechest 33258, FITC, and Cy3 dye were 405/461, 488/520, and 550/570 nm, respectively.

A plan-Apochromat 63× (NA: 1.40) oil objective was used, and magnification was 63 × 1.8. Imaging fields were chosen at random, and Z-sections were optimized for a number of cells. Image size of all images was 1,024 × 1,024 (Pixel), and the zoom was ×1.8.

### Macrophages Stimulation Experiment

RAW264.7 cells (*n* = 6) in 24-well plates were treated with 0.5 ml of LPS (10 ng/ml) for 4 h and then incubated with rACTIN (0.25–1 µg/well) and/or 200–400 ng/well of LP17 peptide (LQVTDSGLYRCVIYHPP), a synthetic polypeptide inhibitor of TREM-1 signaling (11), for an additional 5 h. Then, the cell supernatants were collected for detection of TNF-α concentration with a commercial ELISA kit (eBioscience).

Peritoneal macrophages were isolated and purified from *wt* and *trem1^−/−^* mice (*n* = 6) according to the procedure described before (21). The cells were plated into a 24-well plate at a density of 2 × 10^5^ per well and then incubated with LPS (20 ng/ml) for 4 h, followed by treatment with rACTIN (0.5–1 µg/well) and/or LP17 (100–400 ng/well) for an additional 5 h. Finally, the cell supernatants were collected for detection of TNF-α, IL-6, and MCP-1 concentration with commercial ELISA kits (eBioscience).

### Immunohistochemistry Analysis

The cecal ligation and puncture (CLP)-induced sepsis was performed on three C57BL/6 mice as described before (22). The lung tissues of CLP mice and healthy mice were fixed with 4% paraformaldehyde, and tissue sections were subjected to immunohistochemistry analysis with mouse TREM-1 antibody antigen affinity-purified polyclonal goat IgG (R&D) and rabbit anti-beta actin polyclonal antibody (Proteintech), followed by FITC-conjugated affinipure donkey anti-rabbit IgG (H + L) (Proteintech) and CY3-conjugated affinipure donkey anti-goat IgG (H + L) (Proteintech). After washing five times, all sections were analyzed with a Fluoview™ Fv1000 laser scanning confocal microscope (OLYMPUS) and FV10-ASW3.1 viewer software (OLYMPUS). 405, 488, and 559 nm wavelengths were used, because the Excitation/Emission wavelengths of Hoechest 33258, FITC, and Cy3 dye were 405/461, 488/520, and 550/570 nm, respectively.

The UPLSAPO 100× (NA: 1.40) objective was used, and the zoom was ×1.0, so the magnification was 100 × 1. Imaging fields were chosen at random, and Z-sections were optimized for a number of cells. The image size of all images was 1,024 × 1,024 (pixels).

### Statistical Analysis

The data were analyzed with by unpaired, non-parametric, Mann–Whitney test with GraphPad Prism 6.05, and all the assays were repeated at least three times. A value of *p* < 0.05 served as the threshold of significance.

## Results and Discussion

### A TREM-1 Ligand Expressed on Platelets during Sepsis

**Figure 1:**
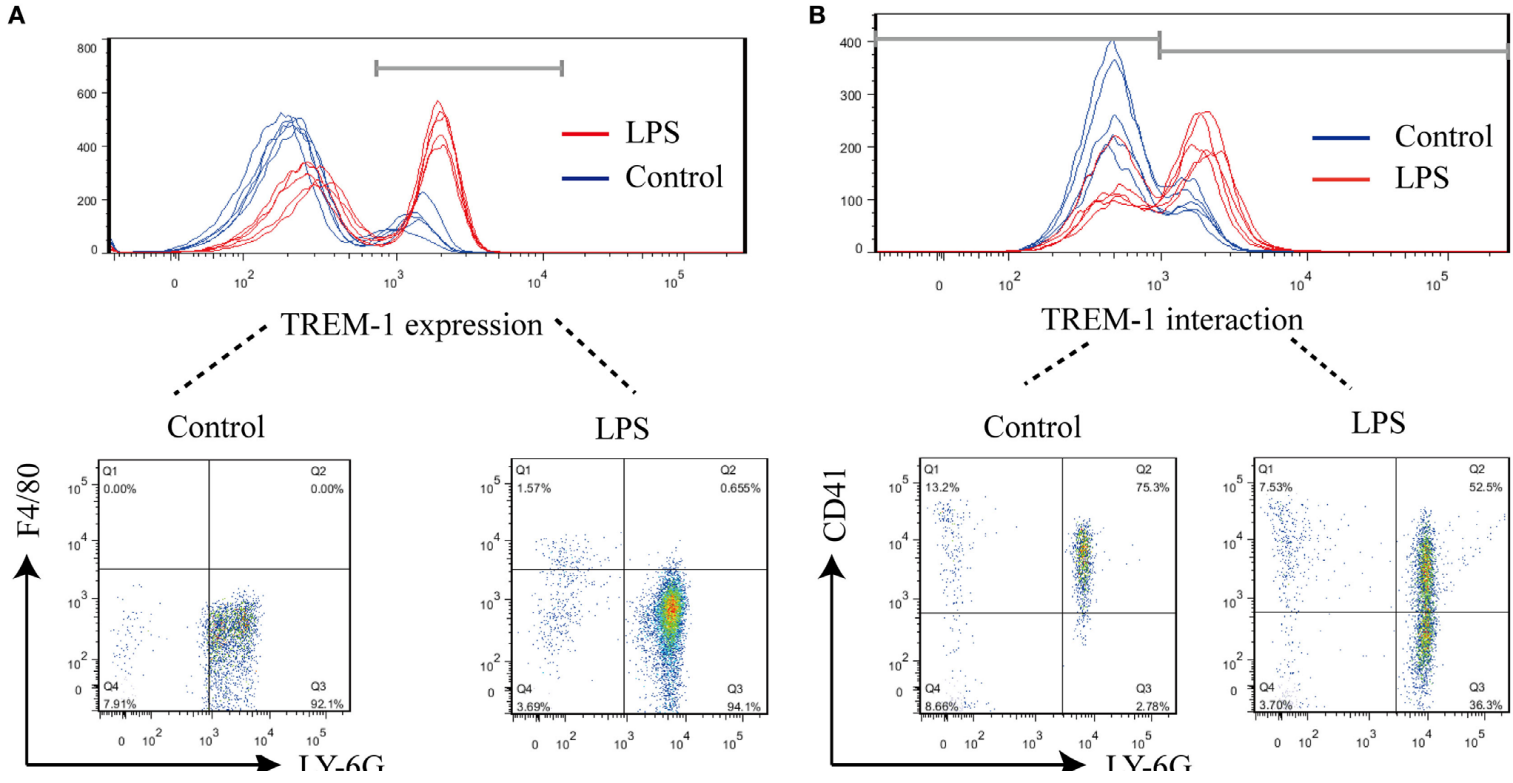
FACS analysis of surface triggering receptor expressed on myeloid cells-1 (TREM-1) and its ligand on cells. Mice (*n* = 5) were inoculated with LPS for 6 h or mock treated with PBS as a control. The blood cells were collected, and red blood cells were removed for analysis of TREM-1 expression or analysis of the distribution of TREM-1-interacting proteins. All experiments were done in triplicate. **(A)** FACS analysis of TREM-1 expression on the cell surface with phycoerythrin (PE)-conjugated rat anti-mouse TREM-1 or PE Rat IgG2a, κ Isotype ctrl Antibody, allophycocyanin-conjugated anti-mouse F4/80, and Percp/cy5.5-conjugated anti-mouse Ly-6G. The signal for TREM-1 was specific (Figure S2 in Supplementary Material), and the cells expressing TREM-1 was further analyzed (Figure S3 in Supplementary Material). **(B)** FACS analysis of the distribution of TREM-1-interacting proteins on cells with Cy5.5-NHS-Ester-labeled recombinant extracellular domain of mouse TREM-1 (rTREM-1), PE/Cy7-conjugated anti-mouse CD41, and PE-conjugated anti-mouse Ly-6G. The cells expressing the ligand for TREM-1 were shown in details (Figure S4 in Supplementary Material).

During LPS-induced sepsis, TREM-1 was induced, and most of TREM-1 was expressed on Ly-6G^+^ cells (95.28 ± 1.11%) (Figure 1A). To further identify which cell expresses the ligand for TREM-1 during sepsis, the rTREM-1, which could interfere with the signaling (19), was labeled with Cy5.5-NHS-Ester to identify which cells provided the ligand. Consistent with previous reports (15), rTREM-1 could recognize CD41^+^ cells during LPS-induced sepsis (Figure 1B). It was very interesting that rTREM-1 could also recognize Ly-6G^+^ cells during sepsis (Figure 1B), and platelets interacting with neutrophils may partially account for this localization (23, 24).

### Identification of Actin As a TREM-1-Interacting Protein on Platelets

**Figure 2:**
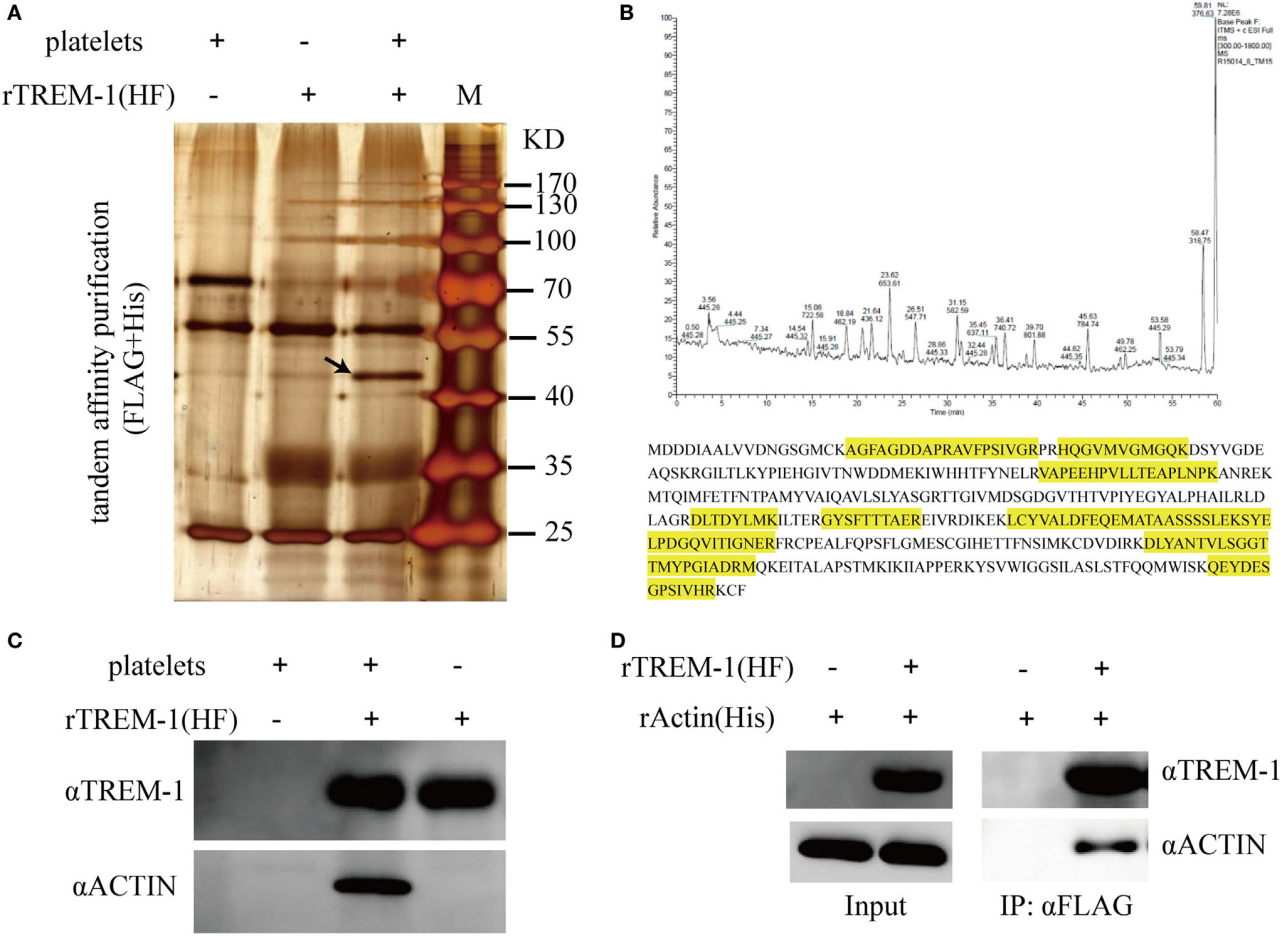
Identification and characterization of actin as a triggering receptor expressed on myeloid cells-1 (TREM-1)-interacting protein on platelets. **(A)** SDS-PAGE gel analysis of the proteins extracted from platelets which were co-purified with recombinant extracellular domain of mouse TREM-1 (rTREM-1) (fusion with FLAG and 6His tag) through nitrilotriacetic acid (NTA) agarose resin and αFLAG-agarose. The interacting protein band was marked by an arrow. **(B)** LC-MS/MS analysis of the protein band extracted from the SDS-PAGE gel **(A)**. The amino acid marked by yellow was identified by MS/MS characterization. **(C)** The purified proteins were further characterized by immunoblotting with antibody against actin or TREM-1. **(D)** The rACTIN purified with NTA agarose resin was incubated with rTREM-1 (fusion with FLAG and 6His tag) purified with NTA agarose resin and then loaded on the αFLAG-agarose. The elution was analyzed by immunoblot with antibody against actin or TREM-1.

Since platelets can provide the ligand for TREM-1 (15), which plays an essential role in inflammatory response in septic syndrome (23, 24), we decided to identify which protein on platelets interacted with the extracellular domain of TREM-1. The total proteins of purified platelets were extracted and loaded onto the NTA agarose resin with rTREM-1 (fusion with 3FLAG and 6His tags) or a control protein. After elution, the proteins were further loaded onto αFLAG-agarose and then analyzed by SDS-PAGE gel (Figure 2A). The specific protein band, which was co-precipitated with rTREM-1, was extracted for LC-MS/MS analysis and identified as actin (Figure 2B). Then, the precipitated proteins were subjected to immunoblot with actin antibody to further confirm that actin was the TREM-1-interacting protein (Figure 2C). To further investigate whether the interaction was direct, purified recombinant actin (rACTIN) was used for precipitation with purified rTREM-1 using αFLAG-agarose. rACTIN could also be directly precipitated with rTREM-1 (Figure 2D), indicating that actin could directly interact with rTREM-1.

### Co-Localization of Actin and TREM-1 during Macrophage Activation

**Figure 3:**
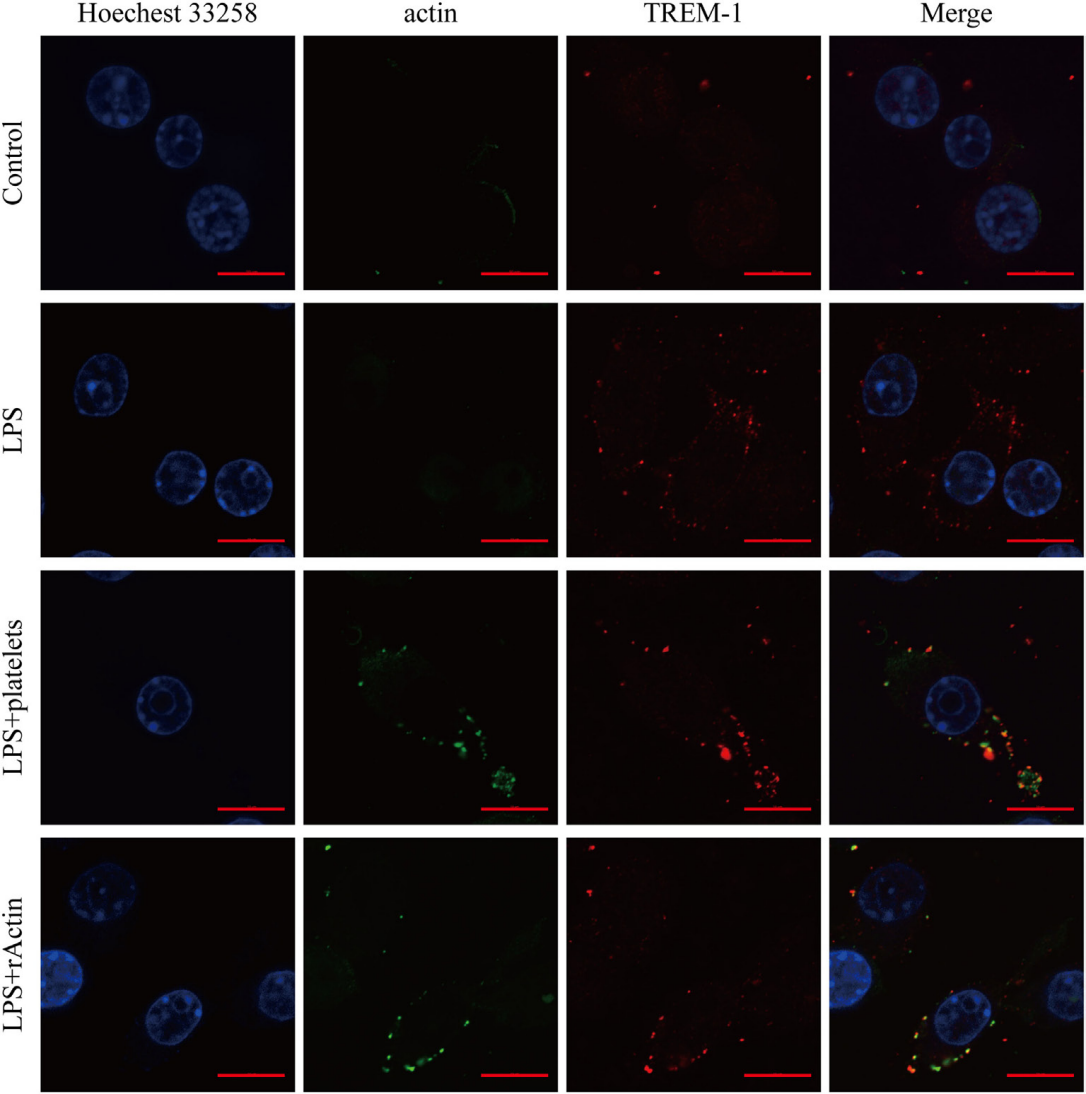
Analysis of the co-localization of triggering receptor expressed on myeloid cells-1 (TREM-1) and actin by laser scanning confocal microscopy. RAW264.7 cells were mock treated with PBS or treated with LPS, platelets, or rACTIN. All cells were fixed and then incubated with mouse TREM-1 antibody antigen affinity-purified polyclonal goat IgG and rabbit anti-beta actin polyclonal antibody, followed by FITC-conjugated affinipure donkey anti-rabbit IgG (H + L) and CY3-conjugated affinipure donkey anti-goat IgG (H + L). After staining the nuclei with Hoechest 33258, the slides were analyzed with a LSM880 with Airyscan laser scanning confocal microscope and ZEN2.3 LITE software. The scale bars in the figure represent 10 µm.

Although actin could interact with TREM-1 *in vitro*, we wanted to further confirm that the interaction was taking place during the activation of TREM-1 signaling. Therefore, we detected whether actin and TREM-1 were co-localized on the surface of myeloid cell during TREM-1 signaling activation. Because platelets could enhance the inflammatory response of mouse macrophages induced by LPS through TREM-1 signaling (15, 19), we aimed to detect whether both proteins were co-localized on LPS-stimulated RAW264.7 cells with platelets or recombinant actin by a confocal microscopy. It was anticipated that TREM-1 would be significantly induced by LPS and that little actin would be found on the surface of macrophages without platelet stimulation (Figure 3). Interestingly, the co-localization of TREM-1 and actin was obvious with further stimulation with rACTIN or platelets (Figure 3), which could enhance the inflammatory response. Because the cells were not treated with a permeabilizing agent, the actin within the cell could not be easily detected, which meant that the co-localization of both proteins was mainly on the cell surface during the activation (Figure 3). These results indicated that platelets could provide surface actins for TREM-1 recognition during the activation of signaling.

### Actin Enhances Macrophage Activation through TREM-1 Signaling

**Figure 4:**
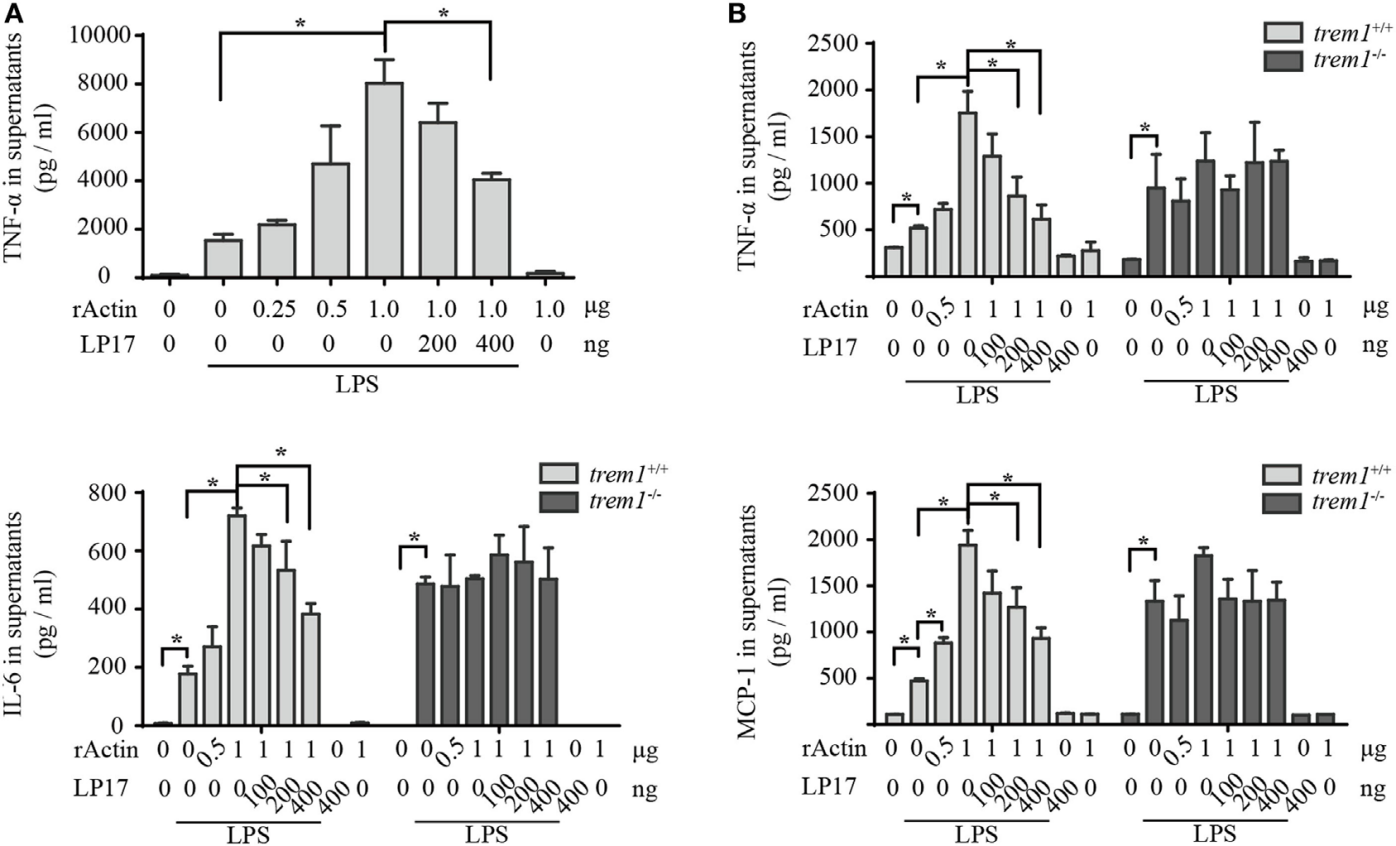
Actin enhances neutrophil activation through triggering receptor expressed on myeloid cells-1 (TREM-1) signaling. **(A)** RAW264.7 cells were treated with LPS for 4 h and further incubated with rACTIN and LP17 for an additional 5 h. Then, the supernatants were collected for detection of TNF-α concentration with a commercial ELISA kit (*n* = 6). **(B)** Peritoneal macrophages isolated from *trem1*^+/+^ or *trem1^−/−^* mice (*n* = 6) were treated with LPS for 4 h and further incubated with rACTIN and LP17 for an additional 5 h. Finally, the supernatants of the cells were collected for the detection of TNF-α, IL-6, and MCP-1 concentration with commercial ELISA kits. The data were expressed the means ± SE of measurement. *p < 0.05.

Because rACTIN could interact with rTREM-1 directly and both proteins showed co-localization during activation, we wanted to further investigate whether the interaction could activate TREM-1 signaling. Similar to the effect of enhancement of inflammatory response by platelets, rACTIN could also enhance TNF-α production by LPS-stimulated RAW264.7 in a dose-dependent manner (Figure 4A). To further address whether the enhancement of inflammatory response by rACTIN was through TREM-1 signaling, we evaluated the effect of LP17, a synthetic polypeptide inhibitor of TREM-1 signaling (11), on enhancement of the inflammatory response by rACTIN. Interestingly, LP17 could inhibit the enhancement of TNF-α induction by rACTIN. This suggested that TREM-1 signaling was required for the enhancement of inflammatory response by rACTIN.

To further confirm that rACTIN enhances LPS-induced inflammatory response through TREM-1 signaling, we compared the enhancement of inflammatory response by rACTIN on LPS-induced peritoneal macrophages isolated from *trem1*^+/+^ or *trem1^−/−^* mice. The rACTIN could enhance *trem1*^+/+^ macrophages to produce TNF-α, IL-6, and MCP-1 in response to LPS stimulation, but the enhancement was not significant for *trem1^−/−^* macrophages (Figure 4B). Furthermore, the enhancement in *trem1*^+/+^ macrophages could be inhibited by LP17 in a dose-dependent manner (Figure 4B). Therefore, the present study indicated that actin could enhance macrophage activation through TREM-1 signaling.

### The Recognition of Actin by TREM-1 during Sepsis

Although the essential roles of either TREM-1 or actin in sepsis have been demonstrated (6, 25), the linking of extracellular actin and TREM-1 activation during sepsis was not previously demonstrated. Because extracellular actin was identified as the ligand for TREM-1 signaling *in vitro*, it prompted us to assess the interaction *in vivo*.

**Figure 5:**
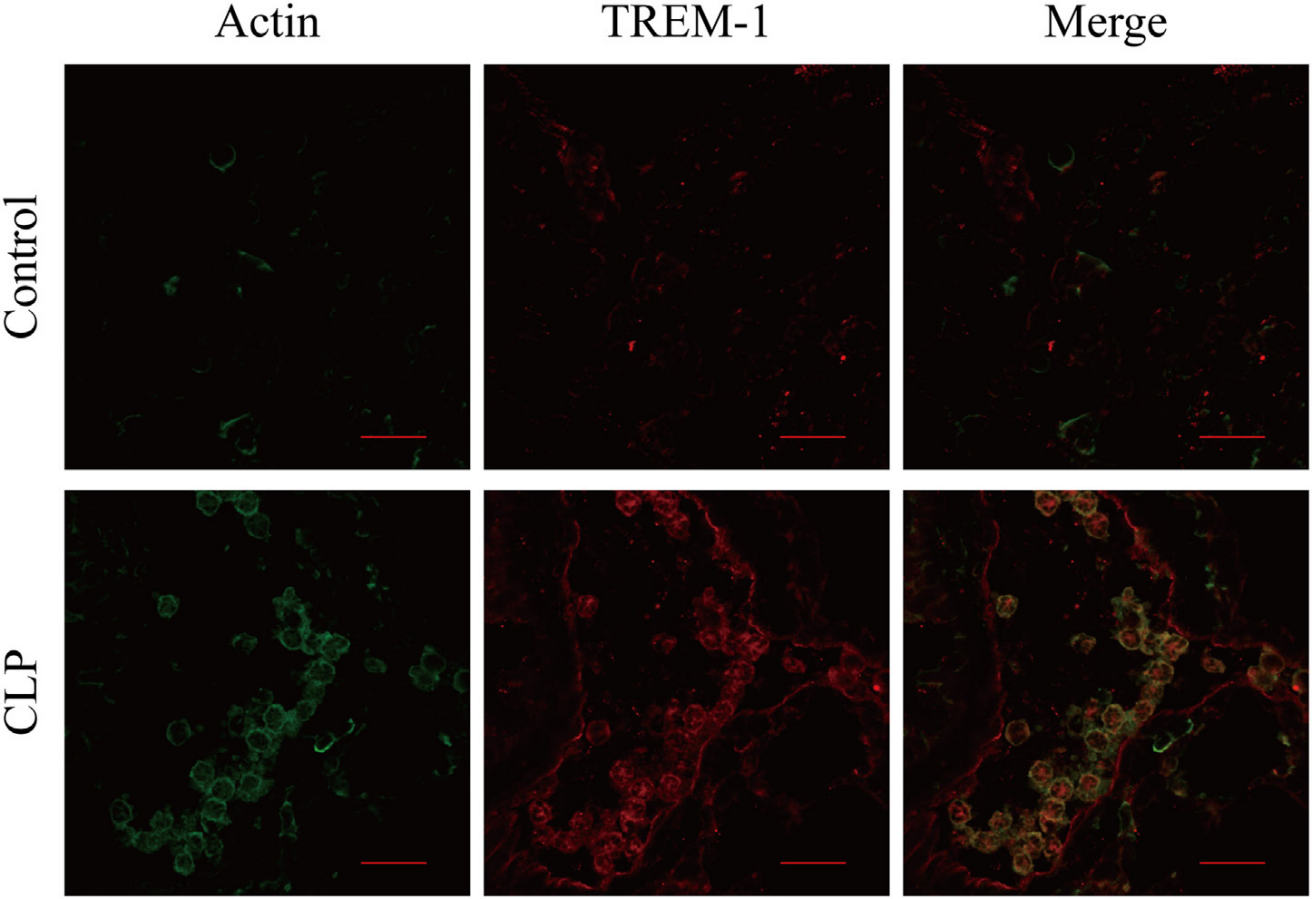
Analysis of the co-localization of triggering receptor expressed on myeloid cells-1 (TREM-1) and actin in lung sections of cecal ligation and puncture (CLP) mice or healthy control mice. The lung sections from CLP mice (*n* = 3) or mock-treated mice (*n* = 3) were stained with mouse TREM-1 antibody antigen affinity-purified polyclonal goat IgG and rabbit anti-beta actin polyclonal antibody, followed by FITC-conjugated affinipure donkey anti-rabbit IgG (H + L) and CY3-conjugated affinipure donkey anti-goat IgG (H + L). The cells were analyzed with Fluoview™ Fv1000 laser scanning confocal microscope and FV10-ASW3.1 viewer software. The scale bars in the figure represent 20 µm.

In the mouse model of CLP-induced sepsis, severe pulmonary inflammation is present. Surface distribution of TREM-1 and actin was observed easily, and more interestingly, almost all the extracellular actin and TREM-1 displayed a similar distribution. However, no obvious signal was detected for extracellular actin or TREM-1 in the control mice (Figure 5). Therefore, the results indicated that TREM-1 is induced and could recognize extracellular actin during sepsis.

Since TREM-1 was identified as an essential regulator of sepsis, identification of the ligand was fascinating because it could benefit the understanding of the contribution of the TREM-1 pathway in sepsis. Platelets can interact with immune cells and contribute to the development of sepsis (23), and a previous study performed by Haselmayer et al. indicated that the natural ligand for TREM-1 was on the surface of platelets (15). The present study further identified actin as a regulator on platelets to activate the signaling. Because actin is a cellular cytoskeleton protein, there is a conflict about whether actin could be distributed on the cell surface. In fact, we did detect the distribution of actin on the surface of platelets with an antibody even on the resting condition (Figure S1 in Supplementary Material) (26). This is similar to the observation by Haselmayer et al., who identified the natural ligand for TREM-1 distributed on the surface of platelets in the resting condition (15). Therefore, platelets did provide surface actin for TREM-1 recognition to activate the signaling.

Cell death-associated proteins (for example, histones) are a kind of very intense DAMP to induce hyperinflammatory responses and are major mediators of death in sepsis (22). In fact, extracellular actin has also been recognized as an important DAMP for a long time and has been associated with a variety of clinical situations, including hepatic necrosis, septic shock, the adult respiratory distress syndrome, and certain disorders of pregnancy (25). However, the mechanism for extracellular actin to cause severe inflammation was not clear. The present study indicated that extracellular actin could active TREM-1 signaling, which provided an explanation for how extracellular actin induces a hyperinflammatory response. The conclusion was further supported by the fact that TREM-1 was induced and showed similar location with extracellular actin during sepsis. Therefore, the present study could provide a link of these two essential mediators of death in sepsis, and it also suggests that the removal of extracellular actin could be a treatment for sepsis (4).

At this point, HMGB1, PGLYRP1, and extracellular actin have been identified as ligands for TREM-1 (12, 18), which means various proteins could active TREM-1 signaling. It was very interesting that all these ligands were previously associated with inflammatory conditions. PGLYRP1 could be induced in response to infection (27); HMGB1 and actin are mainly distributed in the interior of cells in the resting condition and can be released from cells during sepsis (26, 28). This provided an image of how TREM-1 signaling can be activated to control infection or cause severe disease.

In the resting condition, surface actin of platelets would not enhance TREM-1 signaling because the activation of TREM-1 signaling requires the interaction of platelets and neutrophils and is selectin/integrin dependent (15) and because TREM-1 expression is not induced in normal conditions. In contrast, low-level stimulation would induce expression of TREM-1, and the platelets would provide a small quantity of surface actin to activate TREM-1 signaling and induce an inflammatory response. This could be reflected by the finding that a large amount of platelets are required for TREM-1 signaling in *in vitro* experiments. Through these means, the stimulation could enhance inflammatory responses at a controlled level. This might be the reason that TREM-1 signaling is required for some pathogen clearance (19, 29, 30). Unfortunately, if the stimulation is overwhelming, it would significantly induce expression of TREM-1 and cause host cells to die and release actin and HMGB1, which would provide a large number of ligands for activation of TREM-1 signaling and progressive systemic inflammatory responses, resulting in sepsis.

Therefore, the present study identified a new ligand for TREM-1 signaling activation. Importantly, it gave a link between both essential regulators of death in sepsis, although TREM-1 might recognize various molecules for activation.

## Ethics Statement

The study was performed in strict accordance with the Guide for the Care and Use of Laboratory Animals Monitoring Committee of Hubei Province, China, and the protocol was approved by the Committee on the Ethics of Animal Experiments at the College of Veterinary Medicine, Huazhong Agricultural University (permit number: HZAUMO-2015-018). All efforts were made to minimize the suffering of the animals used in the study.

## Author Contributions

LF, LH, CX, WL, LL, SP, YZ, and ZL performed experiments and analyzed the data. MJ contribute to experimental conditions. LH analyzed the data and corrected the manuscript. AZ conceived the project, analyzed the data and prepared the manuscript.

## Conflict of Interest Statement

The authors declare that the research was conducted in the absence of any commercial or financial relationships that could be construed as a potential conflict of interest. The reviewer, CJ, and handling editor declared their shared affiliation, and the handling editor states that the process nevertheless met the standards of a fair and objective review.

## Footnotes

^1^http://www.cdc.gov/sepsis/index.html.

## Supplementary Material

Figure S1 FACS analysis of the distribution of surface actin on platelets. The blood cells from LPS-induced septic mice (*n* = 5) or control mice (*n* = 5) were blocked with donkey sera and rat anti-mouse CD16/32 and then stained with phycoerythrin/Cy7-conjugated anti-mouse CD41 (BioLegend), rabbit anti-beta actin polyclonal antibody (Proteintech), and FITC-conjugated affinipure donkey anti-rabbit IgG (H + L) for analysis of the distribution of surface actin on CD40^+^ cells.

Figure S2 FACS analysis of the specific signal for triggering receptor expressed on myeloid cells-1 (TREM-1) on blood cell. FACS analysis the blood cells form control mice **(A)** and LPS-induced mice **(B)** with phycoerythrin (PE)-conjugated rat anti-mouse TREM-1 or PE Rat IgG2a, κ Isotype ctrl Antibody, allophycocyanin-conjugated anti-mouse F4/80, and Percp/cy5.5-conjugated anti-mouse Ly-6G. The comparison of signal from TREM-1 antibody with signal from isotype control antibody was shown.

Figure S3 FACS analysis of the cells expressing triggering receptor expressed on myeloid cells-1 (TREM-1). **(A)** The blood cells were collected, and red blood cells were removed for FACS analysis. FACS analysis the blood cells form control mice **(B)** and LPS-induced mice **(C)** with phycoerythrin-conjugated rat anti-mouse TREM-1, allophycocyanin-conjugated anti-mouse F4/80, and Percp/cy5.5-conjugated anti-mouse Ly-6G. The cells expressing TREM-1 were further analyzed.

Figure S4 FACS analysis of the cells expressing the ligand for triggering receptor expressed on myeloid cells-1 (TREM-1). The blood cells were collected, and red blood cells were removed for FACS analysis **(A)**. FACS analysis the blood cells form control mice **(B)** and LPS-induced mice **(C)** with Cy5.5-NHS-Ester-labeled recombinant extracellular domain of mouse TREM-1, phycoerythrin (PE)/Cy7-conjugated anti-mouse CD41, and PE-conjugated anti-mouse Ly-6G. The cells expressing the ligand for TREM-1 were shown.
