# Supplementary figures and images for: Identification of Extracellular Actin As a Ligand for Triggering Receptor Expressed on Myeloid Cells-1 Signaling
Source: Front Immunol. 2017 Aug 7;8:917. doi: 10.3389/fimmu.2017.00917 (PMC5545922; doi:10.3389/fimmu.2017.00917)

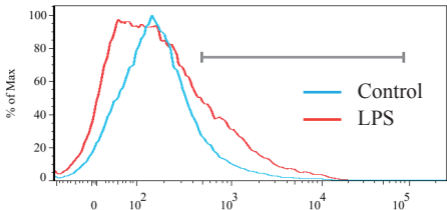

surface actin distribution

Control

LPS

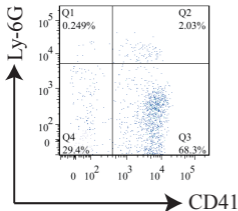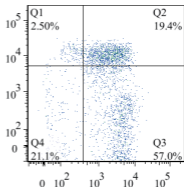

Supplement: Figure S1 — FACS analysis of the distribution of surface actin on platelets. The blood cells from LPS-induced septic mice (n = 5) or control mice (n = 5) were blocked with donkey sera and rat anti-mouse CD16/32 and then stained with phycoerythrin/Cy7-conjugated anti-mouse CD41 (BioLegend), rabbit anti-beta actin polyclonal antibody (Proteintech), and FITC-conjugated affinipure donkey anti-rabbit IgG (H + L) for analysis of the distribution of surface actin on CD40+ cells. [file image_1.pdf]

A

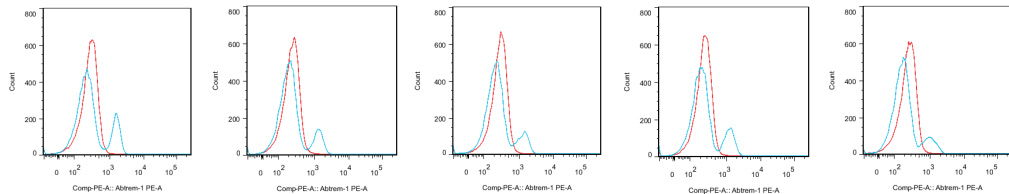

B

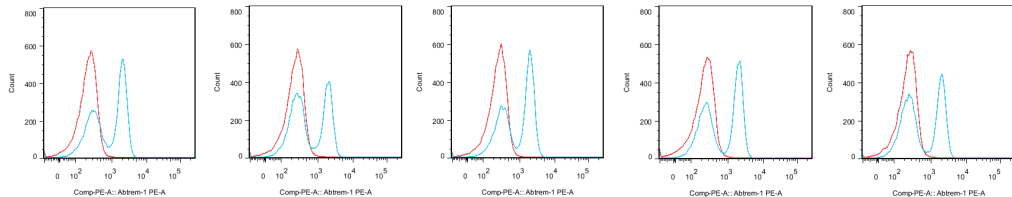

Supplement: Figure S2 — FACS analysis of the specific signal for triggering receptor expressed on myeloid cells-1 (TREM-1) on blood cell. FACS analysis the blood cells form control mice (A) and LPS-induced mice (B) with phycoerythrin (PE)-conjugated rat anti-mouse TREM-1 or PE Rat IgG2a, κ Isotype ctrl Antibody, allophycocyanin-conjugated anti-mouse F4/80, and Percp/cy5.5-conjugated anti-mouse Ly-6G. The comparison of signal from TREM-1 antibody with signal from isotype control antibody was shown. [file image_2.pdf]

A

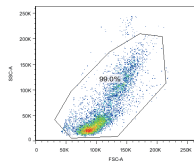

B

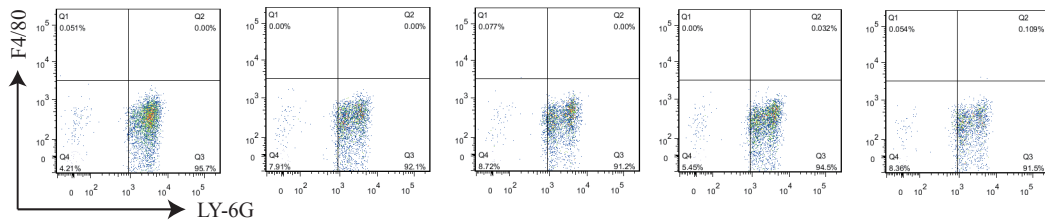

C

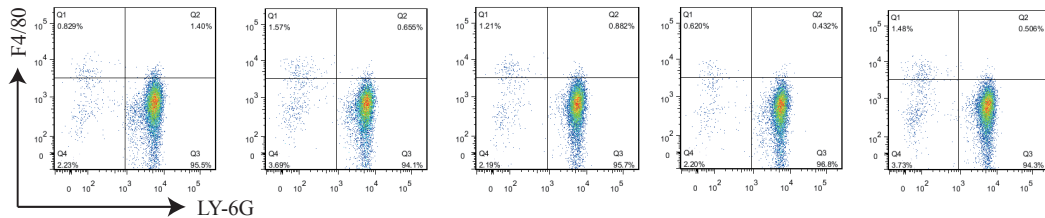

Supplement: Figure S3 — FACS analysis of the cells expressing triggering receptor expressed on myeloid cells-1 (TREM-1). (A) The blood cells were collected, and red blood cells were removed for FACS analysis. FACS analysis the blood cells form control mice (B) and LPS-induced mice (C) with phycoerythrin-conjugated rat anti-mouse TREM-1, allophycocyanin-conjugated anti-mouse F4/80, and Percp/cy5.5-conjugated anti-mouse Ly-6G. The cells expressing TREM-1 were further analyzed. [file image_3.pdf]

A

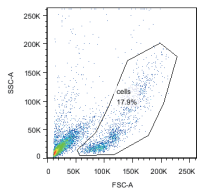

B

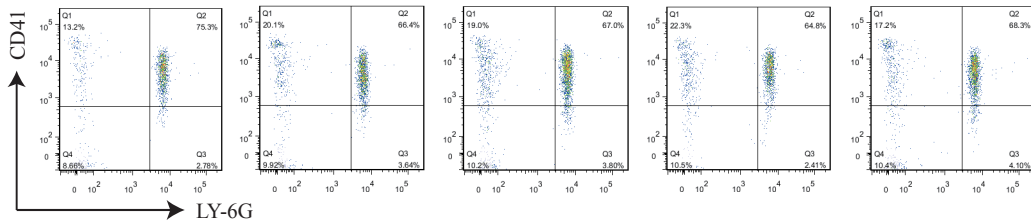

C

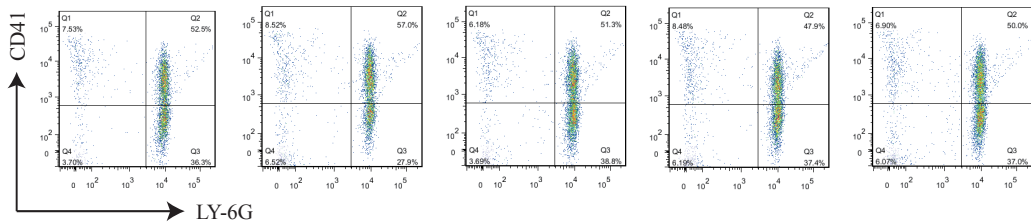

Supplement: Figure S4 — FACS analysis of the cells expressing the ligand for triggering receptor expressed on myeloid cells-1 (TREM-1). The blood cells were collected, and red blood cells were removed for FACS analysis (A). FACS analysis the blood cells form control mice (B) and LPS-induced mice (C) with Cy5.5-NHS-Ester-labeled recombinant extracellular domain of mouse TREM-1, phycoerythrin (PE)/Cy7-conjugated anti-mouse CD41, and PE-conjugated anti-mouse Ly-6G. The cells expressing the ligand for TREM-1 were shown. [file image_4.pdf]
